# The relationships between optimal infant feeding practices and child development and attained height at age 2 years and 6–7 years

**DOI:** 10.1111/mcn.13631

**Published:** 2024-03-07

**Authors:** Lan Mai Tran, Phuong H. Nguyen, Melissa F. Young, Reynaldo Martorell, Usha Ramakrishnan

**Affiliations:** ^1^ Hubert Department of Global Health, Rollins School of Public Health Emory University Atlanta Georgia USA; ^2^ Doctoral Program in Nutrition and Health Sciences, Laney Graduate School Emory University Atlanta Georgia USA; ^3^ Nutrition, Diets, and Health Unit, International Food Policy Research Institute Washington District of Columbia USA; ^4^ Thai Nguyen University of Pharmacy and Medicine Thai Nguyen Vietnam

**Keywords:** attained height, breastfeeding, child development, dietary diversity, early and middle childhood

## Abstract

Limited evidence exists on the long‐term effects of early feeding practices on child growth and development. We examined the relationships between infant feeding practices and child height and development at ages 2 and 6–7 years. We studied 885 mother–child dyads from a randomized controlled trial of preconception supplementation in Vietnam. Early initiation of breastfeeding (EIBF), exclusive breastfeeding (EBF), breastfeeding (BF) duration and minimum dietary diversity (MDD) were assessed using World Health Organization (WHO) guidelines. Child development was assessed by the Bayley Scales of Infant Development‐III at 2 years and the Wechsler Intelligence Scale for Children® – IV at 6–7 years. Child height‐for‐age z‐score (HAZ) was calculated from child height and age. Multivariable regression and structural equation models were used in analyses that controlled for confounding. EIBF and EBF at 6 months occurred in 52% and 62% of children, respectively. Mean breastfeeding duration was 18 months and 83% achieved MDD at 1 year. EIBF was associated with motor (*β* = 0.13, 95% confidence interval [CI]: 0.00, 0.28) and cognitive development at 2 years (*β* = 0.12, 95% CI: −0.01, 0.26), which in turn were positively associated with cognitive development at 6–7 years. EBF was directly associated with development at 6–7 years (*β* = 0.21, 95% CI:0.08, 0.34) whereas motor and cognitive development at 2 years explained 41%–75% of the relationship between EIBF and development at 6–7 years. HAZ at 2 years also mediated 70% of the association between MDD at 1 year and HAZ at 6–7 years. BF duration was not associated with child development and HAZ. Early infant feeding practices, especially EIBF and EBF, have important long‐term implications for optimizing child linear growth and cognition as they begin school.

## Key messages

- Optimal breastfeeding practices during the first few months of life were positively associated with cognitive development in early childhood and school‐aged children.
- While exclusive breastfeeding was directly associated with child cognition at 6–7 years, cognitive development at 2 years mediated the association of early initiation of breastfeeding on cognition at 6–7 years. More research is needed to replicate these findings, explore mechanisms and evaluate the causal relationship.
- Child linear growth in early childhood mediated the association of minimum dietary diversity at 1 year on child height‐for‐age z‐score at 6–7 years.

## Graphical abstract

This study examined the relationships between infant feeding practices during the first year of life and child linear growth and development at ages 2 and 6–7 years. Early feeding practices, especially early initiation of breastfeeding, exclusive breastfeeding and minimum dietary diversity, have important long‐term implications for optimizing child linear growth and cognition as they begin school.

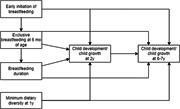

## 1 INTRODUCTION

Optimal infant and young child feeding practices (IYCF) during the first 2 years of life are critical for child development (Perez‐Escamilla et al., 2023; WHO & UNICEF, 2021). Exclusive breastfeeding (EBF) has been associated with better cognition and motor development in childhood and primary school‐aged children (Kramer et al., 2008; Oddy et al., 2003). Longer duration of breastfeeding is also associated with better cognitive development from early childhood through adulthood (Horwood et al., 2001; Huang et al., 2014; Kim & Choi, 2020; Nyaradi et al., 2015; Victora et al., 2005, 2015; Walker et al., 2011). Children and adolescents who were breastfed as babies perform better on intelligence tests and have higher school attendance (Anderson et al., 1999; Horta et al., 2015; Kramer et al., 2008; Victora et al., 2005). Evidence, however, is mixed with some studies showing a modest or even no association between breastfeeding and cognition functioning (Der et al., 2006; Prado et al., 2017; Rochat et al., 2016; Tumwine et al., 2018). In addition, less is known about the role of early initiation of breastfeeding (EIBF) within 1 h of birth which is not only critical for establishing and maintaining breastfeeding practices (Nguyen et al., 2020; Perez‐Escamilla et al., 2023), but has also been shown to protect newborns against infections and neonatal mortality (Hajeebhoy et al., 2014; WHO & UNICEF, 2021).

Dietary diversity in the first 2 years of life has also been positively associated with child development (Larson et al., 2017; Miller et al., 2020; Nyaradi et al., 2015; Prado et al., 2017). Children who had greater dietary diversity at 9–15 months showed higher motor and language scores at 18 months in prospective cohorts in Burkina Faso, Ghana and Malawi (Prado et al., 2017), and had faster reaction times in cognitive performance at 17 years in Australia (Nyaradi et al., 2015). In contrast, results from a cluster‐randomized maternal education trial found a positive association between dietary diversity score at 6–8 months and only fine motor skills at 20–24 months, but no significant associations with other development domains (communication, personal‐social and problem‐solving) (Kakwangire et al., 2021). In addition, results from using pooled cross‐sectional data from the Demographic and Health Survey of 15 low‐ and middle‐income countries (LMICs) in Central America, Caribbean, Asia (West, South and Southeast) and Africa (East, West and Central) show that child minimum dietary diversity was only associated with literacy‐numeracy development, but not with cognitive, socioemotional or physical development among children 36–59 months of age (Bliznashka et al., 2022). These mixed findings of the associations between IYCF practices and child cognition may result from highly variable contexts with inadequate adjustment for multiple aspects of nurturing care, challenges in accurate and standardized assessment of exposures and the development outcomes, and limitations of study design.

Although both breastfeeding and complementary feeding practices are distinct yet inter‐related components of IYCF practices, very few studies have concurrently examined the role of both breastfeeding and complementary feeding practices during the first year of life (Nyaradi et al., 2015; Prado et al., 2017). To our knowledge, no single study in LMICs has simultaneously evaluated the contribution of all four recommended IYCF practices, namely, EIBF, EBF at 6 months, duration of breastfeeding and dietary diversity on child development outcomes in the first 1000 days and beyond.

There is also a limited understanding of the pathways through which optimal IYCF practices might influence child cognition at different time points and whether they operate through growth and development in earlier periods. Most research to date, particularly in LMICs, is cross‐sectional in nature and lacks data on measures of cognitive development at multiple time points. The few studies with multiple cognitive measurements have either explored the predictive value of developmental assessments at previous time points for later measurements of cognition (Girault et al., 2018) or examined associations between breastfeeding and cognitive development at different periods of life together (Kim & Choi, 2020; Oddy et al., 2003). The provision of optimal IYCF practices is the most proximal factor that influences survival, health, growth and development in early childhood (Hurley et al., 2016; Perez‐Escamilla et al., 2023), but major gaps remain in our understanding of the effects of these practices during the school‐age years (Bundy et al., 2017; Keats et al., 2021). Most countries and global programmes remain in the mode of targeting specific issues and age groups in childhood, rather than considering the age continuum and integration of varied periods (Bhutta et al., 2019).

We have a unique opportunity to address these gaps using data on IYCF practices, child growth and development that were measured prospectively from birth through age 6–7 years in a large birth cohort of Vietnamese children. In this study, we aimed to (1) assess the relationships between infant feeding practices and child development and attained height at 2 years and 6–7 years; (2) examine whether cognitive development or attained height at 2 years mediates the effects of IYCF practices on child growth and cognitive development at 6–7 years.

## 2 METHODS

### 2.1 Study design, participants and setting

The study subjects are offspring of women who participated in a randomized controlled trial (RCT) that was designed to evaluate the effects of preconception micronutrient supplementation on maternal and child health outcomes in Vietnam (PRECONCEPT study; NCT: 01665378) (Nguyen et al., 2012). Briefly, 5011 women of reproductive age who intended to get pregnant were recruited and assigned randomly to receive weekly supplements containing either 2800 μg folic acid (FA), 60 mg iron and 2800 μg FA (IFA) or multiple micronutrients (MM) containing the same amount of IFA, from baseline until conception. During pregnancy, women received daily prenatal supplements containing 60 mg iron and 400 μg FA. In the intervention period from 2012 to 2014, a total of 1813 women conceived and delivered 1599 live births. Mother–child dyads (*n* = 1599) were then followed prospectively from delivery to 6–7 years with follow‐up rate of 91.3% (Supporting Information: Figure 1). The inclusion criteria for this secondary data analysis included all live births with data on breastfeeding and complementary feeding practices during the first year of life, as well as anthropometry and development at 2 and 6–7 years of age (*n* = 885). The main reasons for excluding study subjects were the missing data on child development at 6–7 years (*n* = 281), EBF at 6 months (*n* = 179), MDD at 1 year (*n* = 305), anthropometry (*n* = 117) and development (*n* = 138) at age 2 years (Supporting Information: Figure 1).

### 2.2 Child development and cognitive outcomes

We evaluated child development at 2 years of age using the Bayley Scales of Infant Development (BSID) III (Bayley, 2005) which includes cognitive, language and motor subscales. This tool has been translated, validated and adapted for local context, with high internal consistency and high inter‐rater reliability (Nguyen et al., 2017).

Child intellectual development at 6–7 years was assessed using the Wechsler Intelligence Scale for Children® – Fourth Edition (WISC–IV) (Wechsler, 2003) that was translated into Vietnamese and back‐translated by bilingual psychologists and health researchers (Dang et al., 2012). The Adaptation Committee that included four child clinical psychologists from Vietnam and were also familiar with the WISC‐IV, provided overall guidance for the adaption process and revised the tools for cultural appropriateness (Dang et al., 2012). The WISC‐IV was administered by trained paediatricians or researchers with a master's degree in public health.

Data quality was ensured by routine field‐based supervision, monthly staff meetings and regular refresher training sessions. Raw summary scores for each of the subtests were computed for all measures of cognitive development as described in the test manuals and then transformed to composite scores (with mean ± SD of 100 ± 15). Average scores of the cognitive and language domains obtained from BSID‐III were generated as a composite measure of cognitive development at ages 2 years, while Full‐Scale Intelligence Quotient (FSIQ) – a combination of cognition and language domains at 6–7 years was generated from four specific cognitive and language domains (verbal comprehension, perceptual reasoning, working memory and processing speed) that were assessed by the WISC‐IV. All outcome measures were transformed to standardized z‐scores to facilitate comparisons over time within and between individuals. Children at 2 years were classified as normal or various categories of development delay based on recommended cut‐off values for the BSID‐III scores (85 points and above: normal, 70–84 points: mild, 55–69 points: moderate, less than 54: severe) (Celik et al., 2020; Johnson et al., 2014). At 6–7 years, children were categorized into seven groups (very superior: 130 and above, superior: 120–129, high average: 110–119, average: 90–109, low average: 80–89, borderline: 70–79 and extremely low: 69 and bellow) based on the recommended cut‐off of the composite scaled scores in WISC‐IV manual (Weiss et al., 2006).

### 2.3 Child height

Standing height was measured at ages 2 and 6–7 years by trained field staff using standard methods (Cogill, 2003). Collapsible length boards precise to 1 mm were used to measure height. The average of duplicate measurements of height were converted into height‐for‐age z‐scores (HAZ) according to the WHO child growth standards (WHO, 2009, 2010). All children were classified as stunted (HAZ < −2 standard deviation [SD]) or normal height (HAZ ≥ −2 SD) based on the WHO definition (WHO, 2009).

### 2.4 Exposure variables

Breastfeeding and complementary feeding practices were assessed using the standard WHO indicators (WHO & UNICEF, 2021). EIBF data were collected at 1 month and defined as putting a baby to the breast within 1 h of birth. EBF under 6 months was defined as feeding an infant under 6 months of age exclusively with breast milk during the previous day. This indicator was calculated based on the maternal recall of all foods and liquids given to her child in the 24 h before the survey at the follow up visits between 3 and 6 months of age. MDD (defined as the consumption of at least five out of eight food groups during the previous day) was based on maternal recall of all foods and drinks that the children consumed in the previous 24 h at age 12 months. EIBF, EBF and MDD were used as dichotomous variables. Duration of BF was assessed at 24 months follow‐up based on maternal recall of when her child stopped breastfeeding. If children were still breastfeeding at 24 months of follow‐up, the duration of BF was considered equal to child age at the time of survey. The duration of BF was used as a continuous variable.

### 2.5 Confounders

Confounding variables were considered at child, maternal and household levels. Child‐level variables included sex, age, gestational age and birthweight. Maternal level included age at baseline, ethnicity, schooling, occupation, parity and preconception intervention group (FA, IFA and MM). At household level, the quality of the learning environment at home was measured using the Infant/Toddler HOME inventory at 1 year of age (Bradley & Caldwell, 1988); the HOME assesses the quality and quantity of the social, emotional and cognitive support available to a child in the home environment. Household socioeconomic status (SES) index was calculated using a principal components analysis of housing quality and assets that were ascertained at baseline (Gwatkin et al., 2007; Vyas & Kumaranayake, 2006).

### 2.6 Statistical analysis

Descriptive statistics were used to report outcomes and predictors, namely, frequencies and percentages for categorical variables, and means and SDs for continuous variables. We examined differences in characteristics of study participants between the final analytic sample and those lost to follow‐up, using the chi‐squared test (for categorical variables) and t‐test (for continuous variables).

We first evaluated correlations between the different IYCF practices using tetrachoric correlation coefficients and then examined independent associations between each of the IYCF practices and the outcomes at different time points using bivariate and multivariable regression models. Effect modification by the quality of home environment and SES were tested using interaction terms with IYCF practices in the multivariable regression models. We used structural equation models (‘sem’ command in Stata) to evaluate the pathways through which the IYCF practices might contribute to child development and estimated the direct and indirect associations between the IYCF practices and child development, after adjusting for all potential confounders. Separate pathways through attained HAZ and development outcomes at 2 years were evaluated based on the results of the multivariable regression models. We assessed model fit based on the model chi‐square (less than 0.05), comparative fit index value (greater than 0.90) and root mean square error of approximation value (less than 0.06) (Hooper et al., 2008). The indirect effects, calculated by multiplying coefficients for each path, allowed us to compare the relative strength of each path.

We also conducted sensitivity analysis, in which we imputed the missing values in IYCF practices using both maximum likelihood with missing value (mlmv option in sem command) (Enders & Bandalos, 2001) and multiple imputation method (multiple imputation by chained equation – MICE, 15 imputations) (Azur et al., 2011) under the assumption that the data were missing at random.

All analyses were conducted using Stata v17 (StataCorp). A significant level of 0.05 was used for all statistical tests.

### 2.7 Ethical approval

The study was approved by the Ethical Committee of the Institute of Social and Medicine Studies in Vietnam and Emory University's Institutional Review Board, Atlanta, Georgia, USA. The trial was registered in the US Clinical Trials registry. Written informed consent was obtained from all study participants.

## 3 RESULTS

The final analytic sample included 885 mother–child dyads with data on IYCF practices, anthropometry and development at 2 and 6–7 years. The description of the final analytic sample is presented in Table 1. Mean cognitive development score at 2 years was 101.4 points (SD 9.5), and FSIQ score at 6–7 years was 88.2 points (SD 12.2). At 2 years, cognitive and motor delay (mild and moderate/severe groups) were found in less than 5% and 1% children, respectively, whereas at 6–7 years, 4.6% children were classified as extremely low and 17% in borderline groups. About a fifth of children were stunted at 2 years, and 10% were stunted at 6–7 years. Breastfeeding was suboptimal with half of children having EIBF in the first hour after birth and 62% having EBF at 6 months of age. At age 1 year, more than 80% of children achieved MDD. Mean breastfeeding duration was 18 months and breastfeeding prevalence steeply declined during the second year of life from 97% at 12 months to 63% at 18 months, and only 6% at 24 months. The final analytic sample was similar in most characteristics to those missing data, except that children in the analytic sample were slightly older (Supporting Information: Table 1).

**Table 1:** Characteristics of the final analytic sample.

	Mean ± SD/%		Mean ± SD/%
	*n* = 885		*n* = 885
Maternal characteristics		Child growth	
Age at baseline, years	26.0 ± 4.4	HAZ at 2 years, z‐score	−1.3 ± 0.9
Minority ethnic, %	50.3	Stunting at 2 years, %	22.2
Maternal education, years	9.7 ± 2.8	HAZ at 6–7 years, z‐score	−0.8 ± 0.9
Work as farmers, %	81.0	Stunting at 6–7 years, %	9.9
Parity, *n*	2.0 ± 0.4	Child development	
Preconception intervention, %		*Age 2 years*	
MM	32.8	Cognitive development, score	101.4 ± 9.5
IFA	31.8	Normal, %	95.7
FA	35.4	Mild, %	4.1
Child characteristics		Moderate, %	0.2
Gestational age, weeks	39.2 ± 2.0	Severe, %	0
Preterm, %	9.5	Motor development, score	106.5 ± 12.2
Birthweight, g	3082.5 ± 420.8	Normal, %	99.1
Low birthweight, %	4.9	Mild, %	0.8
Female, %	47.2	Moderate, %	0.1
Child age at 2 years study, month	24.4 ± 0.4	Severe, %	0
Child age at 6–7 years study, month	77.1 ± 3.7	*Age 6–7 years*	
Household characteristics		Full‐scale IQ, score	88.2 ± 12.2
Home environment at 12 months	63.3 ± 8.0	Very superior	0
Socioeconomic status index	0.0 ± 0.9	Superior	0.6
IYCF practices		High average	3.5
Early initiation of breastfeeding, %	51.6	Average	38.6
Exclusive breastfeeding at 6 months of age, %	61.7	Low average	35.7
Minimum dietary diversity at 1 year, %	82.8	Borderline	17.0
Breastfeeding duration, month	17.9 ± 3.2	Extremely low	4.6
Breastfeeding duration ≥12 months, %	97.4		
Breastfeeding duration ≥18 months, %	63.1		
Breastfeeding duration ≥24 months, %	6.1		

Abbreviations: FA, folic acid; HAZ, height‐for‐age z‐score; IFA, iron and folic acid; IYCF, infant and young child feeding; MM, multiple micronutrients.

EIBF was positively associated with motor (*β* = 0.14, 95% CI: 0.00, 0.28) and cognitive development at age 2 years (*β* = 0.13, 95% CI: −0.01, 0.26) (Table 2). MDD at 1 year was also positively associated with motor and cognitive development (*β* = 0.16, 95% CI: −0.02, 0.34 and *β* = 0.22, 95% CI: 0.04, 0.39, respectively) at age 2 years, but these associations were attenuated in adjusted models. The association between MDD at 1 year and HAZ at 2 years was positive, but not statistically significant (*β* = 0.15, 95% CI: −0.01, 0.31), and this association remained after controlling for other confounders. No associations were observed between EBF or breastfeeding duration with either child HAZ or development at 2 years.

**Table 2:** Association between infant feeding practices and child development and attained size at 2 years.^a^

	Motor development		Cognitive development		HAZ	
	Unadjusted	Adjusted^b^	Unadjusted	Adjusted^b^	Unadjusted	Adjusted^b^
	*n* = 885	*n* = 861	*n* = 885	*n* = 861	*n* = 885	*n* = 861
Infant feeding practices	*β* (95% CI)	*β* (95% CI)	*β* (95% CI)	*β* (95% CI)	*β* (95% CI)	*β* (95% CI)
Early initiation of breastfeeding	0.13 [−0.01,0.27]	0.14* [0.00,0.28]	0.11 [−0.02,0.25]	0.13 [−0.01,0.26]	−0.00 [−0.13,0.12]	−0.00 [−0.12,0.12]
Exclusive breastfeeding at 6 months of age	0.08 [−0.06,0.22]	0.07 [−0.07,0.21]	0.05 [−0.09,0.19]	0.04 [−0.10,0.17]	0.04 [−0.09,0.16]	0.03 [−0.09,0.15]
Breastfeeding duration	−0.01 [−0.03,0.01]	−0.02 [−0.04,0.00]	−0.01 [−0.02,0.02]	−0.01 [−0.03,0.01]	−0.02 [−0.04,0.00]	−0.02 [−0.04,0.00]
Minimum dietary diversity at 1 year of age	0.16 [−0.02,0.34]	0.10 [−0.08,0.28]	0.22* [0.04,0.39]	0.15 [−0.03,0.32]	0.15 [−0.01,0.31]	0.14 [−0.01,0.30]

Abbreviations: CI, confidence interval; HAZ, height‐for‐age z‐score.

^a^ Separate models were run for each of the four infant feeding practices.

^b^ Model adjusted for maternal age, ethnicity, education, occupation, parity, child age, sex, gestational age, birthweight, home quality environment, household socioeconomic status and preconception treatment group.

*p < 0.05.

EBF at 6 months of age was significantly associated with cognitive development at 6–7 years (*β* = 0.20, 95% CI: 0.07, 0.33) (Table 3). However, no associations were observed for other BF practices. MDD at 1 year was positively associated with HAZ at 6–7 years in both unadjusted (*β* = 0.16, 95% CI: −0.01, 0.32) and adjusted models (*β* = 0.14, 95% CI: −0.02, 0.31), but these associations were not statistically significant (*p* > 0.05). There was also no evidence of effect modification by home quality or SES.

**Table 3:** Association between infant feeding practices and child development and attained size at 6–7 years.^a^

	FSIQ		HAZ	
	Unadjusted *β*	Adjusted *β* b	Unadjusted *β*	Adjusted *β* b
	*n* = 885	*n* = 861	*n* = 885	*n* = 861
Infant feeding practices	*β* (95% CI)	*β* (95% CI)	*β* (95% CI)	*β* (95% CI)
Early initiation of breastfeeding	0.01 [−0.12, 0.14]	0.04 [−0.09, 0.17]	−0.05 [−0.18, 0.07]	−0.03 [−0.16, 0.09]
Exclusive breastfeeding at 6 months of age	0.19** [0.05, 0.33]	0.20** [0.07, 0.33]	0.03 [−0.10, 0.16]	0.02 [−0.10, 0.15]
Breastfeeding duration	−0.01 [−0.03, 0.01]	−0.01 [−0.03, 0.01]	−0.01 [−0.03, 0.01]	−0.01 [−0.03, 0.01]
Minimum dietary diversity at 1 year of age	0.10 [−0.07, 0.28]	0.03 [−0.14, 0.20]	0.16 [−0.01, 0.32]	0.14 [−0.02, 0.31]

Abbreviations: CI, confidence interval; FSIQ, Full‐Scale Intelligence Quotient; HAZ, height‐for‐age z‐score.

^a^ Separate models were run for each of the four infant feeding practices.

^b^ Model adjusted for maternal age, ethnicity, education, occupation, parity, child age, sex, gestational age, birthweight, home quality environment, household socioeconomic status and preconception treatment group.

**p < 0.01.

**Figure 1:**
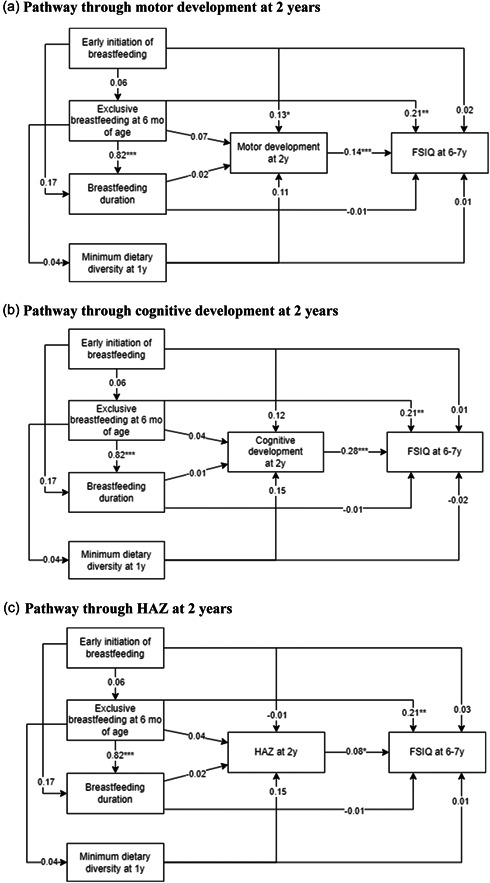
Pathways of optimal infant feeding practices and child development at 6–7 years through child development and HAZ outcomes at 2 years.^a^ (a) Pathway through motor development at 2 years. (b) Pathway through cognitive development at 2 years. (c) Pathway through HAZ at 2 years. *p < 0.05, **p < 0.01, ***p < 0.001. ^a^Model adjusted for maternal age, ethnicity, education, occupation, parity, child age, sex, gestational age, birthweight, home quality environment, household socioeconomic status and preconception treatment group. FSIQ, Full‐Scale Intelligence Quotient; HAZ, height‐for‐age z‐score.

Three distinct pathways through which optimal IYCF practices might affect child cognition at 6–7 years were examined. First, we found that EIBF was directly associated with motor development at 2 years (*β* = 0.13, 95% CI: 0.00, 0.27) which in turn was associated with cognitive development at 6–7 years (*β* = 0.14, 95% CI: 0.08, 0.21) (Figure 1a). Similarly, EIBF was associated with cognition at 2 years (*β* = 0.12, 95% CI: −0.01, 0.25) which in turn was significantly associated with cognition at age 6–7 years (*β* = 0.28, 95% CI: 0.22, 0.34) (Figure 1b). The indirect path mediated by motor and cognitive development at 2 years explained 40%–75% of the relationship between EIBF and cognitive development at 6–7 years. EBF was also associated directly with cognitive development at 6–7 years (*β* = 0.21, 95% CI: 0.08, 0.34) and indirectly through motor and cognitive development at 2 years. This indirect path, however, was small and not significant. MDD at 1 year was positively associated with HAZ at 2 years (*β* = 0.15, 95% CI: −0.01, 0.30) which in turn was significantly associated with cognitive development at 6–7 years (*β* = 0.08, 95% CI: 0.01, 0.16) (Figure 1c). However, both the direct and indirect associations through HAZ at 2 years were not statistically significant.

**Figure 2:**
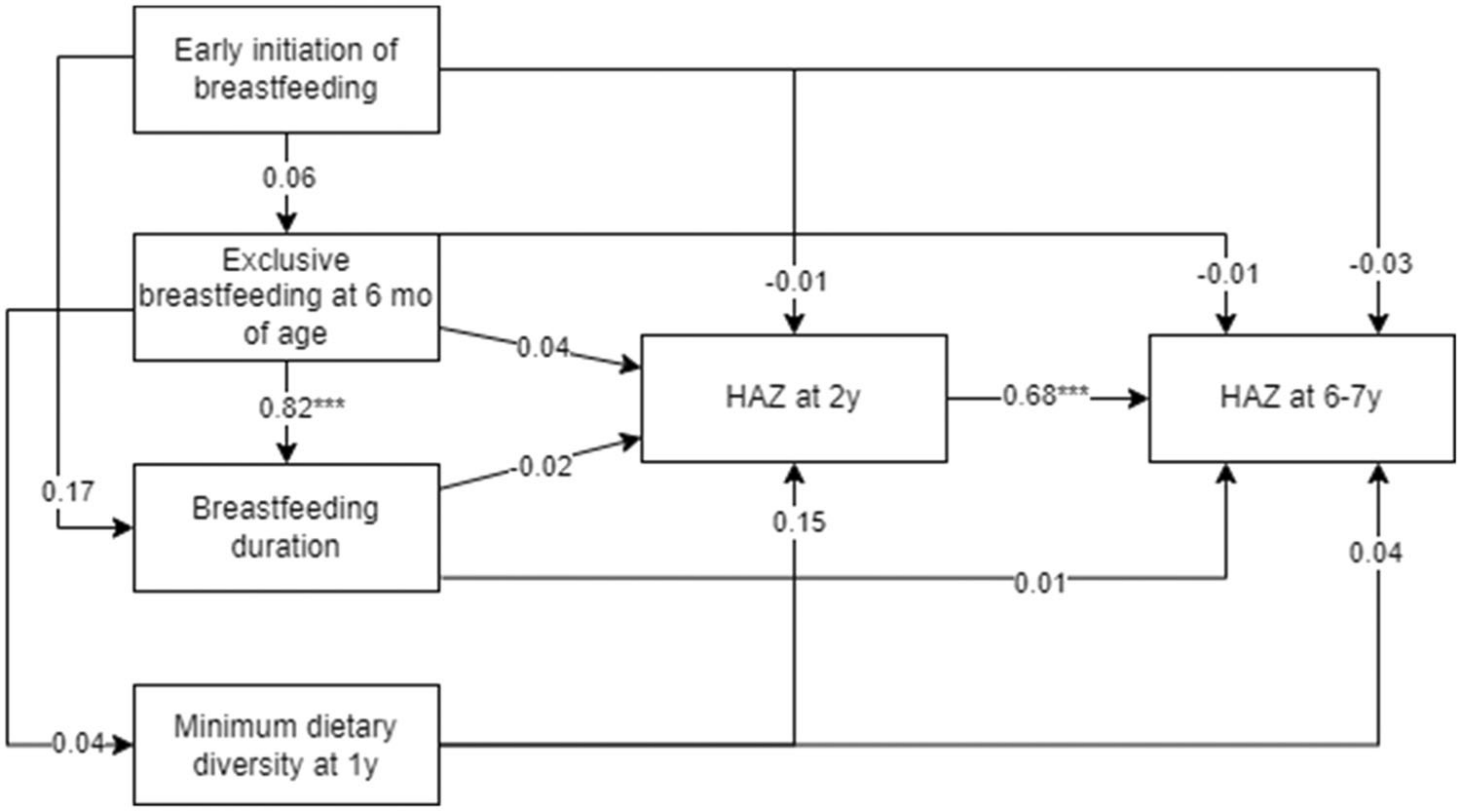
Pathways of optimal infant feeding practices and HAZ at 6–7 years through HAZ at 2 years.^a^ *p < 0.05, **p < 0.01, ***p < 0.001. ^a^Model adjusted for maternal age, ethnicity, education, occupation, parity, child age, sex, gestational age, birthweight, home quality environment, household socioeconomic status and preconception treatment group. HAZ, height‐for‐age z‐score.

The results of the pathways of IYCF practices to child HAZ at 6–7 years show that HAZ at 2 years mediated the associations of MDD and HAZ at 6–7 years and the indirect association of MDD on HAZ at 6–7 years through HAZ at 2 years accounted for 70% of total association (Figure 2). There were, however, no significant associations between BF practices and HAZ at any time point.

The sensitivity analysis in the samples with imputed values shows similar results in the association between IYCF practices and child growth and development (Supporting Information: Figure 2).

## 4 DISCUSSION

Optimal feeding practices during the first year of life were positively associated with child development and growth outcomes at 2 and 6–7 years. Both EIBF and EBF were positively associated with cognitive development while MDD was associated with child linear growth. The difference in motor development score at 2 years between EIBF and non‐EIBF children was nearly two points which represents a small effect size (0.14 SD). Motor and cognitive development at 2 years also mediated 40%–75% of the relationship between EIBF and cognitive development at 6–7 years. Children who were EBF at 6 months of age had FSIQ scores at 6–7 years nearly three points higher (0.2 SD) compared to those who were not EBF. HAZ at 2 years mediated 70% of the association between MDD and HAZ at 6–7 years.

Our findings on the long‐term positive association of BF on cognitive functioning are consistent with previous findings (Anderson et al., 1999; Horta et al., 2015; Kramer et al., 2008; Oddy et al., 2003; Victora et al., 2015). A meta‐analysis of 16 observational studies with subjects from 1 to 15 years revealed an increase of 2.6–3.4 IQ points in BF children compared to non‐BF counterparts after controlling for maternal intelligence, home stimulation and other confounding factors (Horta et al., 2015). Although most of the evidence is from observational studies, a randomized controlled trial in Belarus (PROBIT) showed that the experimental group of EBF had 5.9 points higher in FSIQ at 6.5 years than the control group (Kramer et al., 2008). This cognitive benefit could potentially be explained by several mechanisms. One possible mechanism is through dynamic and interactional nature of breastfeeding practices (Bode et al., 2020). More specifically, BF may contribute to better cognitive development through its promotion of greater maternal responsiveness to infants, psychological bonding of the mother–infant dyad and verbal exchange during this practice (Yang et al., 2018). The physical and/or emotional act of BF has been hypothesized to lead to permanent physiologic changes that accelerate neurocognitive development (Kramer et al., 2008). Most interestingly, it is also possible that the earlier initiation of BF results in increased interaction between mother and infant, which in turn promotes cognitive development. Another possible mechanism that may explain the higher cognitive score among breastfed children relates to the unique composition of breast milk, including various nutritional, non‐nutritional, immune and biological signalling molecules (Christian et al., 2021; Kramer et al., 2008). There is a higher concentration and absorption of both the nutritive and nonnutritive components of human milk such as long‐chain polyunsaturated fatty acids and insulin‐like growth factor I during infancy. Infancy is also a period of rapid neurodevelopment. The co‐occurrence of these two phenomenon might also explain the benefits of early breastfeeding on cognition (Cockburn, 2003; Deoni et al., 2013; Guesnet & Alessandri, 2011; Philipps et al., 1995; Thompson & Nelson, 2001), suggesting that increased consumption of human milk in the first 6 months is critical for cognitive development (Schuchardt et al., 2010). While gaps in knowledge of the underlying mechanisms remain, our findings clearly support the importance of promoting breastfeeding, especially EIBF and EBF during the first few months of life, for optimizing cognitive function.

EIBF is strongly associated with prelacteal practices, influencing the likelihood of EBF and prolonged BF (Nguyen et al., 2020; Perez‐Escamilla et al., 2023), which supports the additive effect of the four IYCF practices (reflecting the combination of EIBF and EBF) on cognitive development that we observed in our study (Supporting Information: Tables 2 and 3). Our study also showed significant indirect associations of EIBF and EBF on intellectual functioning at 6–7 years through both motor and cognitive development outcomes at 2 years, revealing the pathways through which optimal IYCF practices affect child cognition at different time points. These pathways were aligned with noted theory and evidence of both reciprocal relations and dependency among motor and cognitive skills from infancy through early childhood (Girault et al., 2018; Kim et al., 2018). However, the significant association between cognition and the separate EIBF and EBF practices still raises the question of whether colostrum and/or EIBF play a role in child development as it does in protecting newborns against infections and reducing neonatal mortality. Although EIBF has been associated with a reduced risk of language impairment among 4‐ to 11‐year‐old children (Diepeveen et al., 2017), more research is needed to replicate these findings and evaluate the causal relationship. More interestingly, our study illustrated a direct association between EBF and cognitive development at 6–7 years, implying the role of intense maternal bonding and stimulation strengthened by EBF practice.

Although several studies have shown positive associations between BF duration and reduced risk of infectious and chronic diseases, and/or improved child growth and cognitive development (Horwood et al., 2001; Huang et al., 2014; Kim & Choi, 2020; Lee et al., 2016; Meek et al., 2020; Nyaradi et al., 2015; Victora et al., 2005; Wallenborn et al., 2021), we did not find any associations between BF duration and child growth and development. This inconsistency may be due to the variation in BF duration. The mean duration of BF was much longer (18 months) in our study sample and almost all children (97.4%) were still being breastfed at 12 months compared to other studies where only ~30% of children were breastfed more than 12 months (Kim & Choi, 2020; Wallenborn et al., 2021). As a result, most studies compared cognitive development among children who were breastfed for >3 months (Kim & Choi, 2020), 4 months (Nyaradi et al., 2015), 8 months (Horwood et al., 2001) or 9 months (Lee et al., 2016; Victora et al., 2005) to those who had shorter BF duration or those who did not receive breast milk at all. With the current recommendation of WHO to prolong BF for 24 months, additional research is required to better understand the benefits of BF beyond the first year of life for cognition.

Several methodological issues need to be considered while evaluating the reasons for the inconsistency in the literature on the effects of BF on cognition (Der et al., 2006; Prado et al., 2017; Prado et al., 2017; Rochat et al., 2016; Tumwine et al., 2018). First, the studies were conducted in a variety of contexts at different time points from 18 months through 30 years. Each context is distinctive in multiple aspects of nurturing care that provide the ongoing care, guidance, protection and support for children to attain their developmental potential, leading to the differences in the effect size of BF in different contexts. Second, the accuracy and standardization of methods used to measure both BF practices and cognitive functioning are varied and make it difficult to compare between studies. For example, cognition has been assessed using several measurement tools such as the Development Milestones Checklist, Kilifi Development Inventory, MacArthur‐Bates Communicative Development Inventory, WISC, etc. that are administered in different ways (interviewing subjects/caregivers, observing subjects or computerized self‐administered test). Third, differences in study design contribute to mixed findings. To date, most of the evidence comes from cross‐sectional studies, some others come from retrospective and prospective cohorts, and only one community randomized controlled study (Bliznashka et al., 2022; Horta et al., 2015; Kramer et al., 2008; Larson et al., 2017; Oddy et al., 2003; Victora et al., 2015). Taken together, our findings, which are based on a well‐studied prospective cohort, enrich the evidence of the effects of breastfeeding on child development in early childhood.

We also found that MDD was associated with attained height but not with cognitive development. Several previous studies also showed that MDD was significantly associated with attained height at early childhood, but not at primary school age (Arimond & Ruel, 2004; Bwenge Malembaka et al., 2019; Onyango et al., 2014). Although our findings of the effects of MDD on attained height are similar to previous findings, the lack of association with cognitive outcomes contrasts with previous work that demonstrated positive findings (DiGirolamo et al., 2020; Nyaradi et al., 2015; Prado et al., 2017). This might be explained by the high homogeneity of dietary diversity in our sample (83% of children achieved MDD at 1 year). In addition, in our study setting, delayed introduction of diverse diets and inability to ensure MDD during the second year of life were associated with impaired linear growth in early childhood (Duong et al., 2023), suggesting that maintaining MDD in the second year of life may be important for both child growth and cognition. Furthermore, attained height at 2 years was strongly associated with attained height at 6–7 years while its association with cognitive development at 6–7 years was much smaller. The difference in magnitude of these associations might explain why HAZ at 2 years successfully mediated the association of MDD on HAZ, but not on FSIQ at 6–7 years. The positive association of MDD in early childhood with later HAZ found in this study, however, adds new and important information and strengthens the scientific evidence supporting recommendations of delivering prospective interventions across each life stage to achieve the highest gains in nutritional status (Victora et al., 2021).

The key strengths of our study are the prospective study design and the availability of data that allowed us to evaluate pathways from early feeding practices to child growth and development. Evidence of long‐term benefits of BF for improved cognitive functioning has been criticized due to residual confounding by socioeconomic status, parental education and quality of home environment that may explain the association between BF and performance in intelligence tests (Bradley & Corwyn, 2002; Papp, 2014). We were able to adjust for maternal education which was strongly correlated with maternal IQ and still found significant effects of BF similar to the findings of Horta's meta‐analysis (Horta et al., 2015). We also observed no interaction between IYCF practices and SES as well as the home environment. However, we are not be able to rule out the influence of other individual and household determinants of child cognition that are part of health, nutrition, safety/security, learning and relationships as documented in the nurturing care framework for early childhood development (Black & Trude, 2019; Black et al., 2022; Black, 2019; WHO, UNICEF, & WB, 2018). Early learning opportunities, including both attendance and the quality of the early childhood learning environment in pre‐school and kindergarten, have been linked to positive child development (Hurley et al., 2016). Although most of our study children (97%) attended public kindergarten at the appropriate age, only ~11% attended public pre‐school. We, however, lacked data on the quality of the learning environment and social interactions in these settings, as well as other risk factors such as deficiencies in key micronutrients, namely iodine, iron and zinc, exposure to nicotine, and heavy metals such as lead during early childhood that can be negatively associated with child development (Walker et al., 2007).

In conclusion, feeding practices during the first year of life play an important role in both growth and development during early childhood and primary school age. BF practices during infancy directly and indirectly influenced child development in early and middle childhood. Diet quality was also positively associated with child growth, which in turn influenced cognitive functioning. Interventions to improve IYCF should focus on promoting EIBF as this is a key first step to improve other optimal BF practices during the first year of life which in turn benefit child growth and development. Integrated Early Childhood Development programmes that include interventions to improve child feeding, responsive care and early learning support should be strengthened to stimulate learning readiness to help children reach their full potential. Further research examining the mechanisms as well as other outcomes related to neurocognitive function, including attention, educational attainment and lifestyle behaviours, would shed additional light on the long‐term neurocognitive benefits of breastfeeding (Yang et al., 2018).

## AUTHOR CONTRIBUTIONS

Lan Mai Tran, Phuong H. Nguyen, Melissa F. Young and Usha Ramakrishnan performed the research. Phuong H. Nguyen, Usha Ramakrishnan, Melissa F. Young and Reynaldo Martorell designed the research. Lan Mai Tran, Phuong H. Nguyen and Usha Ramakrishnan analysed and interpreted data. Lan Mai Tran, Usha Ramakrishnan, Phuong H. Nguyen, Reynaldo Martorell and Melissa F. Young wrote the paper. All authors have read and approved the final manuscript.

## CONFLICT OF INTEREST STATEMENT

The authors declare no conflict of interest.

## Supporting information

Supporting information.

## Data Availability

The data that support the findings of this study are available from the corresponding author upon reasonable request.
