# Supplementary material for: The relationships between optimal infant feeding practices and child development and attained height at age 2 years and 6–7 years
Source: Matern Child Nutr. 2024 Mar 7;20(3):e13631. doi: 10.1111/mcn.13631 (PMC11168365; doi:10.1111/mcn.13631)
Supplement: Supplementary file 1 — Supporting information. [file MCN-20-e13631-s001.docx]

**Supplemental Figure 1: Flow diagram of participant** **progress throughout the study**

**
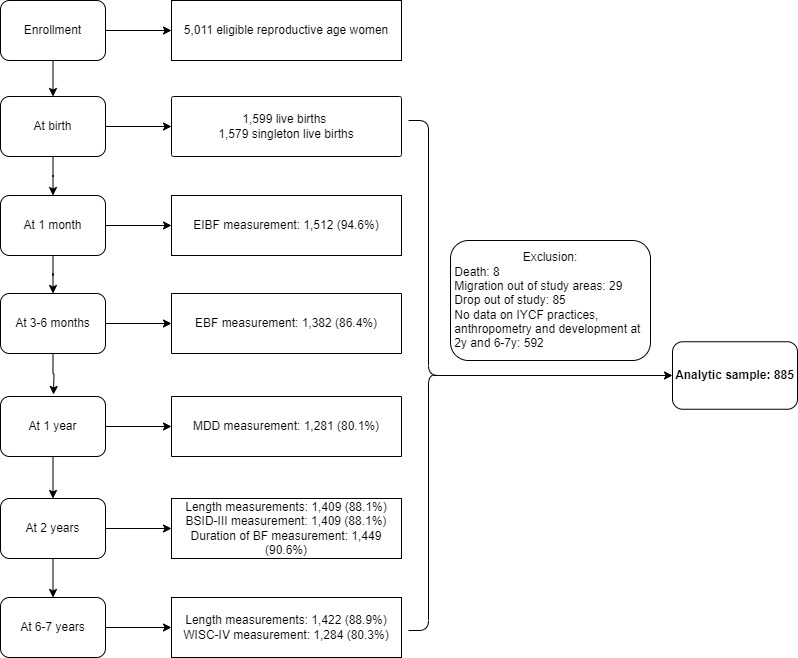
**

EIBF: Early initiation of breastfeeding, EBF: Exclusive breastfeeding, MDD: Minimum dietary diversity, IYCF: Infant and young child feeding, BSID-III: Bayley Scales of Infant Development Third Edition, WISC-IV: Wechsler Intelligence Scale for Children®—Fourth Edition

**Supplemental table 1: Comparison of participants’ characteristics in the final analytic sample and those missing data at follow-up^1^**

|  | **Analytic sample**  ***(n= 885)*** | **Missing data**  ***(n= 714)*** |
| --- | --- | --- |
|  | **Mean** ± S**D/ %** | **Mean** ± **SD/ %** |
| **Maternal characteristics** |  |  |
| Age at baseline, *y* | 26.0 ± 4.4 | 25.8 ± 4.2 |
| Minority ethnic, *%* | 50.3 | 48.3 |
| Maternal education, y | 9.7 ± 2.8 | 9.6 ± 3.0 |
| Work as farmers, *%* | 81.0 | 78.1 |
| Parity, n | 2.0 ± 0.4 | 2.0 ± 0.4 |
| **Preconception intervention, %** |  |  |
| MM | 32.8 | 32.9 |
| IFA | 31.8 | 33.1 |
| FA | 35.5 | 34.0 |
| **Child characteristics** |  |  |
| Gestational age, *wk* | 39.2 ± 2.0 | 39.1 ± 2.0 |
| Preterm, *%* | 9.2 | 11.1 |
| Birth weight, *g* | 3082.5 ± 420.8 | 3084.7 ± 467.1 |
| Low birth weight, *%* | 4.9 | 4.5 |
| Female, *%* | 47.2 | 52.2 |
| Child age at 2 years study, month | 24.4 ± 0.4 | 24.2 ± 0.8 |
| Child age at 6-7 years study, month | 77.1 ± 3.7*** | 75.8 ± 5.7 |
| **Household characteristics** |  |  |
| Home environment at 12 months | 63.3 ± 8.0 | 63.2 ± 8.6 |
| Socio-economic status |  |  |
| Lowest | 34.9 | 31.9 |
| Average | 34.6 | 31.9 |
| Highest | 30.5 | 36.2 |

*** P< 0.001

MM: Multiple micronutrients, IFA: Iron and Folic acid; FA: Folic acid.

**Supplemental table 2: Association between infant feeding practices with child attained size and development at 2 years^1^**

| **Feeding practices** | **Frequency** | **Motor development** | | **Cognitive development** | | **HAZ** | |
| --- | --- | --- | --- | --- | --- | --- | --- |
|  |  | **Unadjusted** | **Adjusted^2^** | **Unadjusted** | **Adjusted^2^** | **Unadjusted** | **Adjusted^2^** |
|  |  | **n=885** | **n=861** | **n=885** | **n=809** | **n=885** | **n=861** |
|  | **%** | **β (95% CI)** | **β (95% CI)** | **β (95% CI)** | **β (95% CI)** | **β (95% CI)** | **β (95% CI)** |
| 1 practice | 5.7 | Ref. | Ref. | Ref. | Ref. | Ref. | Ref. |
| 2 practices | 24.1 | 0.25 [-0.07,0.57] | 0.24 [-0.08,0.56] | 0.10 [-0.19,0.39] | 0.09 [-0.18,0.36] | 0.12 [-0.19,0.44] | 0.13 [-0.18,0.43] |
| 3 practices | 44.9 | 0.32^*^ [0.01,0.63] | 0.28 [-0.02,0.59] | 0.15 [-0.13,0.42] | 0.15 [-0.11,0.41] | 0.29 [-0.01,0.59] | 0.27 [-0.02,0.56] |
| 4 practices | 25.4 | 0.42^**^ [0.11,0.74] | 0.39^*^ [0.08,0.70] | 0.14 [-0.15,0.43] | 0.12 [-0.15,0.39] | 0.31 [0.00,0.62] | 0.28 [-0.02,0.58] |

^1^A combination of four infant feeding practices: early initiation of breastfeeding, exclusive breastfeeding at 6 months, continued breastfeeding and minimum dietary diversity at 1 year. 1 practice = any one practice out of four listed practices, 2 practices = any two practices out of four listed practices, 3 practices = any three practices out of four listed practices, 4 practices = all four listed practices.

^2^Model adjusted for maternal age, ethnicity, education, occupation, parity, child age, sex, gestational age, birth weight, home quality environment, household socio-economic status, and preconception treatment group.

HAZ: Height-for-age z-score.

**Supplemental table 3: Association between infant feeding practices with child attained size and development at 6-7 years^1^**

| **Feeding practices** | **FSIQ** | | **HAZ** | |
| --- | --- | --- | --- | --- |
|  | **Unadjusted** | **Adjusted^2^** | **Unadjusted** | **Adjusted^2^** |
|  | **n=885** | **n=861** | **n=885** | **n=861** |
|  | **β (95% CI)** | **β (95% CI)** | **β (95% CI)** | **β (95% CI)** |
| 1 practice | Ref. | Ref. | Ref. | Ref. |
| 2 practices | -0.01 [-0.32,0.30] | -0.01 [-0.32,0.29] | 0.11 [-0.18,0.41] | 0.13 [-0.15,0.42] |
| 3 practices | 0.09 [-0.21,0.39] | 0.09 [-0.20,0.38] | 0.14 [-0.14,0.42] | 0.16 [-0.11,0.44] |
| 4 practices | 0.21 [-0.09,0.52] | 0.21 [-0.08,0.51] | 0.12 [-0.17,0.41] | 0.14 [-0.15,0.42] |

^1^A combination of four infant feeding practices: early initiation of breastfeeding, exclusive breastfeeding at 6 months, continued breastfeeding and minimum dietary diversity at 1 year. 1 practice = any one practice out of four listed practices, 2 practices = any two practices out of four listed practices, 3 practices = any three practices out of four listed practices, 4 practices = all four listed practices.

^2^Model adjusted for maternal age, ethnicity, education, occupation, parity, child age, sex, gestational age, birth weight, home quality environment, household socio-economic status, and preconception treatment group.

FSIQ: Full-Scale Intelligence Quotient, HAZ: Height-for-age z-score.

**Supplemental Figure 2: Pathways of optimal infant feeding practices and child development and HAZ at 6-7 years through child development and HAZ outcomes at 2 years using sample with imputed missing values^1^.**

1. **Pathway to child development at 6-7y through motor development at 2 years
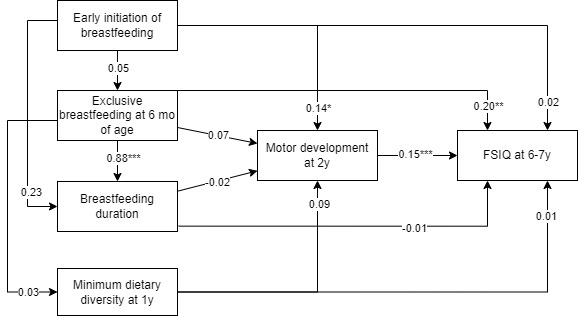
**
2. **Pathway to child development at 6-7y through cognitive development at 2 years
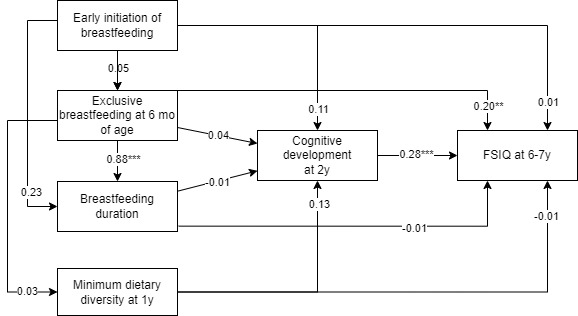
**
3. **Pathway to child development at 6-7y through HAZ at 2 years**

**
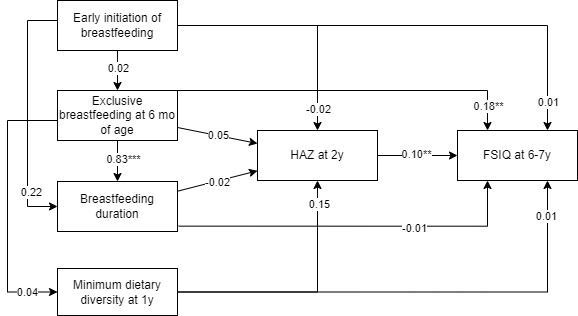
**

1. **Pathway to child HAZ at 6-7y through HAZ at 2 years
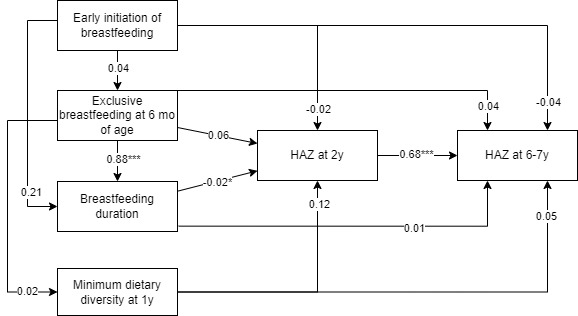
**

*p<0.05, **p<0.01, ***p<0.001

^1^Model adjusted for maternal age, ethnicity, education, occupation, parity, child age, sex, gestational age, birth weight, home quality environment, household socio-economic status, and preconception treatment group.

HAZ: Height-for-age z-score.
